# The enteric nervous system and the musculature of the colon are altered in patients with spina bifida and spinal cord injury

**DOI:** 10.1007/s00428-016-2060-4

**Published:** 2017-01-06

**Authors:** Marjanne den Braber-Ymker, Martin Lammens, Michel J.A.M. van Putten, Iris D. Nagtegaal

**Affiliations:** 1 Department of Pathology, Radboud University Medical Center, PO Box 9101, 6500 HB Nijmegen, The Netherlands; 2 Department of Pathology, Antwerp University Hospital, University of Antwerp, Edegem, Belgium; 3 MIPRO, University of Antwerp, Antwerp, Belgium; 4 Department of Clinical Neurophysiology, MIRA, Institute for Biomedical Technology and Technical Medicine, University of Twente, Enschede, The Netherlands; 5 Department of Neurology and Clinical Neurophysiology, Medisch Spectrum Twente, Enschede, The Netherlands

**Keywords:** Colon, Immunohistochemistry, Intestinal motility, Neuromuscular disease, Spina bifida, Spinal cord injury

## Abstract

Neurogenic bowel dysfunction occurs in a large percentage of adult patients with spina bifida (SB) and spinal cord injury (SCI), significantly affecting their quality of life. Although bowel motility is autonomously regulated by the enteric nervous system (ENS), disruption of the modulation of the ENS by extrinsic innervation as present in many patients with SB and SCI might lead to motility disorders. In order to gain insight in the pathophysiology, we studied histological changes of the neuromuscular structures in the colon of SB and SCI patients. Archival colon tissue blocks from SB (*n* = 13) and SCI (*n* = 34) patients were collected nationwide in The Netherlands and compared with control samples (*n* = 16). Histological (semiquantitative) evaluation of the ENS, the network of interstitial cells of Cajal (ICC), and the muscularis propria was performed using hematoxylin and eosin, periodic acid Schiff, and elastic von Gieson staining, and immunohistochemistry with antibodies against HuC/D, calretinin, S100, CD117, α-smooth muscle actin, and desmin. Compared to controls, SB and SCI patients showed neuronal loss and decreased nerve fiber density in the myenteric plexus. Lower nerve fiber density was significantly more often found in patients with severe bowel dysfunction. Other major findings were loss of ICCs around the myenteric plexus and fibrosis in the longitudinal muscle layer. Altered histology of the ENS may explain abnormal intestinal motility in SB and SCI patients. Furthermore, loss of myenteric nerve fibers (including enteric glial cells) may play a major role in the development of severe motility complaints.

**Electronic supplementary material:**

The online version of this article (doi:10.1007/s00428-016-2060-4) contains supplementary material, which is available to authorized users.

## Introduction

Spina bifida (SB) and spinal cord injury (SCI) are both disorders of the central nervous system (CNS). Neurogenic bowel dysfunction is very common following SB and SCI, in approximately 40% and 42–81% of adult patients, respectively [1–3]. In 39% of SCI patients, colorectal dysfunction has a variable but sometimes significant impact on social activities or quality of life [2, 4]. Main symptoms include loss of bowel control (fecal incontinence), constipation, and lack of bowel movements [1, 5–8]. The occurrence of these complications depends at least partly on the type and the level of the lesion and the time since the injury [3, 9].

Motility and secretion in the colon are controlled by both intrinsic and extrinsic innervation. The intrinsic enteric nervous system (ENS) has two major parts: the submucosal plexus and the myenteric plexus, which are located between the circular and longitudinal layers of the muscularis propria. The submucosal plexus mainly controls mucosal secretion and blood flow, while the myenteric plexus is primarily involved in coordination of motility patterns [10, 11]. One of the local reflexes regulated by the ENS is the peristaltic reflex, which is responsible for normal propulsion of bowel contents. Neurons involved in this reflex include (sensory) intrinsic primary afferent neurons (IPANs), interneurons and motor neurons. Motor neurons give excitatory and inhibitory signals via the interstitial cells of Cajal (ICCs) to smooth muscle cells [12]. Enteric ganglia contain both neurons and glial cells, which play a supportive role to the neurons. Enteric glial cells are increasingly recognized as important for regulatory functions in the gut, such as the control of motility [13]. Although ICC networks are found in different layers of the bowel wall, the ICC network around the circumference of the myenteric plexus may play a major role in peristalsis [14–16].

The ENS functions largely independently of the CNS, although extrinsic input from the sympathetic and parasympathetic nerves modulates the activity of the ENS [17, 18]. Parasympathetic fibers facilitate contraction of the colon musculature, and sympathetic fibers inhibit colon motility [19]. Hence, disruption of the extrinsic nerve fibers as in SB and SCI has a major effect on ENS activity and results in abnormal motor function [20]. The resulting intestinal dysfunction is called neurogenic bowel [4]. This clinically well-known phenomenon is rarely studied on morphological level [21].

Morphological analysis of motility disorders is difficult, since a wide variety of morphological and functional alterations of the ENS, ICCs, and smooth muscle tissue may result in gastrointestinal neuromuscular diseases (GINMDs). Despite the recent introduction of a classification of these alterations by an International Working Group [22], the correlation between clinical manifestations and morphological findings remains challenging due to the lack of systematic studies. Therefore, we performed this nationwide study to evaluate the histopathology of the colon in SB and SCI. This is the first systematic study investigating morphological alterations in these patient groups, which might provide additional insight into the underlying pathophysiology of bowel dysfunction in SB and SCI. Since the etiology of SB and SCI varies, different histological features might be expected in both groups.

The aim of this study was to describe histological alterations of the neuromuscular apparatus in the colon following SB and SCI. Systematic (semiquantitative) assessment involved the main relevant neuromuscular structures of the bowel (ENS, ICCs, smooth muscle). We applied the proposed international guidelines on histological reporting for gastrointestinal neuromuscular pathologies [22, 23].

## Methods

### Subjects

Patients were selected using the nationwide network and registry of histopathology and cytopathology in The Netherlands (PALGA database), which registers all pathology reports since 1991 [24]. Subsequently, archived formalin-fixed paraffin-embedded tissue samples of colon segments were obtained from patients with SB (*n* = 13) or SCI (*n* = 34) who underwent surgical resection of part of the bowel. In seven patients, the site of the removed segment was not indicated; in seven patients, this was the proximal bowel and in 33 patients the distal bowel.

Control segments of the colon (*n* = 16) were obtained from patients who underwent right-sided hemicolectomy for non-obstructive colon carcinoma. Control patients showed no evidence of gastrointestinal motility disorders. Tissue blocks were obtained at a distance of ≥10 cm from the tumor, and these were histologically confirmed as normal.

The study was approved by the local ethics committee (reference number 2014-1256). Samples were obtained in accordance with the Code of Conduct of the Federation of Medical Scientific Societies in the Netherlands [25].

### Tissue preparation

Sections were cut from formalin-fixed paraffin-embedded full-thickness tissue blocks for conventional histology or immunohistochemistry. In the patient groups, most sections were transversal (SB *n* = 10; SCI *n* = 22), but some were longitudinal (SB *n* = 1; SCI *n* = 5) or tangled (SB *n* = 3; SCI *n* = 8). In the control group, only transversely oriented sections were available.

Sections were deparaffinized by standard protocol in xylene, rehydrated in an ethanol series, and rinsed in tap water.

### Histological staining

Sections of 4 μm were used for hematoxylin and eosin (H&E) and periodic acid Schiff (PAS) staining. Elastic von Gieson (EVG) staining was performed on 6-μm sections. Tissues were stained by standard protocols in a Medite TST 30 stainer (Klinipath, Duiven, The Netherlands).

### Immunohistochemistry

Immunohistochemical staining was performed on 4-μm sections. Antibodies, suppliers, and dilutions are listed in Table 1.

**Table 1:** Primary antibodies used for immunohistochemistry

Antibody	Clone	Manufacturer	Dilution	Antigen retrieval
Calretinin	5A5	Novocastra	1:25	EDTA pH 9, 10 min at 96 °C
HuC/D	16A11	Molecular Probes	1:600	Sodium citrate 10 mM (pH 6.0), 30 min at 100 °C
S100	Polyclonal	DAKO	1:10,000	EDTA pH 9, 10 min at 96 °C
CD117	YR145	Immunologic	1:200	None
α-Smooth muscle actin (α-SMA)	1A4	Sigma	1:7500	None
Desmin	33	Biogenex	1:100	Citrate pH 6.7, 30 min at 100 °C

For HuC/D staining, antigen retrieval was performed in sodium citrate (pH 6) at 100 °C for 30 min. Subsequently, endogenous peroxidase was blocked with 3% hydrogen peroxide in PBS for 20 min. Sections were then rinsed in PBS and incubated with primary antibody anti-HuC/D at 4 °C overnight. After washing in PBS, sections were incubated for 30 min with a secondary antibody (Powervision poly-HRP anti Ms/Rb/Rt IgG, Immunologic, Duiven, The Netherlands) at room temperature. Sections were finally rinsed in PBS and immunoreactivity was developed with PowerDAB (Immunologic) for 7 min at room temperature. Subsequently, sections were rinsed in tap water, counterstained with hematoxylin, rinsed in tap water, dehydrated in 100% ethanol and xylene, and mounted with Permount.

The other immunohistochemical staining reactions were performed in an automated LabVision Autostainer 480 (Klinipath, Duiven, The Netherlands). First, the method used for antigen retrieval depended on the antibody (Table 1). Subsequently, endogenous peroxidase was blocked with 3% hydrogen peroxide in methanol for 10 min. Sections were incubated with primary antibody for 60 min. Subsequently, the sections were incubated with Powervision poly-HRP anti Ms/Rb/Rt for 30 min, followed by staining with PowerDAB for 7 min and counterstaining with hematoxylin for 1 min. All incubations were performed at room temperature.

Tissue blocks containing different tissue types were used as controls, with known staining patterns for both positive and negative stained tissues.

### Microscopic analysis

Sections were evaluated blind to diagnosis and were then compared with those of the controls. The histology of the bowel wall was examined by H&E. PAS was used to verify the presence or absence of polyglucosan inclusion bodies in the muscularis propria. The presence or absence of fibrosis was assessed in the submucosa, muscularis propria, and myenteric plexus on EVG-stained sections (Fig. 3c).

Immunohistochemically stained sections were assessed by semiquantitative scoring using visual analysis to evaluate systematically the neuronal structures and smooth muscle layers.

The presence of neurons in ganglia was analyzed on HuC/D- and calretinin-stained sections. The number of neurons in relation to the present plexus was estimated in HuC/D sections as follows: Firstly, the distribution of the neuronal network was evaluated on S100-stained sections. Subsequently, the number of neurons per ganglion was estimated in the HuC/D staining in relation to this neuronal network and scored as 0, no neurons; 1, low neuronal density; and 2, high neuronal density (Suppl. Fig. 1). The calretinin-stained sections were comparably rated: 0, no neurons; 1, on average less than one neuron per neuronal structure; and 2, minimal one neuron per neuronal structure (Suppl. Fig. 2).

S100 was used to assess the distribution of nerve fibers (including nuclei of glial cells) in the submucosa, the myenteric plexus, and both muscle layers of the muscularis propria. The degree of distribution was scored as follows: 0 (no/low density) and 1 (high density of positive fibers) (Suppl. Fig. 3).

The network of ICCs surrounding the myenteric plexus was estimated on CD117-stained sections as described earlier [26]. The percentage of the circumference which is covered by CD117-positive cells was rated from 0 to 100% in 10% increments. Thus, a percentage of 0% represented no positive cells around the ganglia and in sections estimated as 100% the ganglia were completely surrounded by CD117-positive cells. Staining of mast cells was used as internal positive control.

α-Smooth muscle actin (α-SMA) and desmin staining were used to assess the muscularis layers. Staining intensities of circular and longitudinal muscle layers were classified in two grades: 0 (no/weak) and 1 (strong staining intensity) (Suppl. Fig. 4). Immunoreactivity within blood vessel walls and muscularis mucosae acted as internal reference for α-SMA and desmin, respectively (grade 1).

Estimation of interobserver variation for the evaluation of staining resulted in interobserver agreement of α-SMA “almost perfect” (*n* = 17), HuC/D submucosal plexus “fair” (0.444), and myenteric plexus “substantial” (0.609) (*n* = 15). Of cases with multiple slides, the slide with the lowest overall scoring results was used for analysis.

### Statistical analysis

Categorical variables were described by percentages. Differences between categorical variables were assessed by the chi-square test (likelihood ratio, exact *p* values compared with the control group). Continuous variables were presented as means ± standard deviation (SD). The Kruskal-Wallis test with post hoc pairwise comparisons using the Dunn-Bonferroni approach (SPSS procedure) was performed to compare the CD117 scores between groups, computing adjusted *p* values corrected for multiple testing. A *p* value of 0.05 was considered significant. Data were analyzed by the IBM SPSS Statistics 20 Software (SPSS Inc., Chicago, IL, USA) and GraphPad Prism version 5.00 for Windows (GraphPad Software, San Diego, CA, USA).

## Results

SB and SCI patients underwent surgery for several reasons, not limited to complaints that might be related to motility disorders. Therefore, the SB and SCI groups were each divided into two subgroups: first, a subgroup with known (severe) motility disorders which constituted the indication for bowel resection (from here referred to as “symptomatic patients”; SB: *n* = 7, SCI: *n* = 21) and, second, a subgroup in which motility disorders were not listed in the anonymized pathology reports, although possibly present in less severe form (from here referred to as “asymptomatic patients”; SB: *n* = 6, SCI: *n* = 13). The latter subgroup included various indications for resection: carcinoma, diverticulitis, and inflammatory bowel disease (Crohn’s disease, ulcerative colitis). Table 2 shows patient characteristics. An overview of the major results is given in Suppl. Table 1.

**Table 2:** Patient characteristics

		Control (*n* = 16)	SB		SCI	
			Symptomatic (*n* = 7)	Asymptomatic (*n* = 6)	Symptomatic (*n* = 21)	Asymptomatic (*n* = 13)
Gender, *n* (%)	Male	6 (37.5%)	6 (85.7%)	0 (0%)	10 (47.6%)	7 (35.8%)
Age, years (mean ± SD)		62.1 ± 12.9	15.1 ± 14.1	31.0 ± 9.8	55.4 ± 13.6	58.5 ± 14.8
Orientation cross section, *n* (%)	Transversal	16 (100%)	4 (57.1%)	5 (83.3%)	14 (66.7%)	7 (53.8%)
	Longitudinal	0 (0%)	1 (14.3%)	0 (0%)	4 (19.0%)	1 (7.7%)
	Oblique	0 (0%)	2 (28.6%)	1 (16.7%)	3 (14.3%)	5 (38.5%)

*SB* spina bifida, *SCI* spinal cord injury, *SD* standard deviation

H&E staining confirmed the normal histomorphology of control specimens.

### Neuronal changes

#### Submucosal plexus

The presence of neurons relative to nerve fibers in the submucosal plexus was evaluated by HuC/D (all neurons) and calretinin staining (part of intrinsic primary afferent neurons, IPANs).

**Fig. 1:**
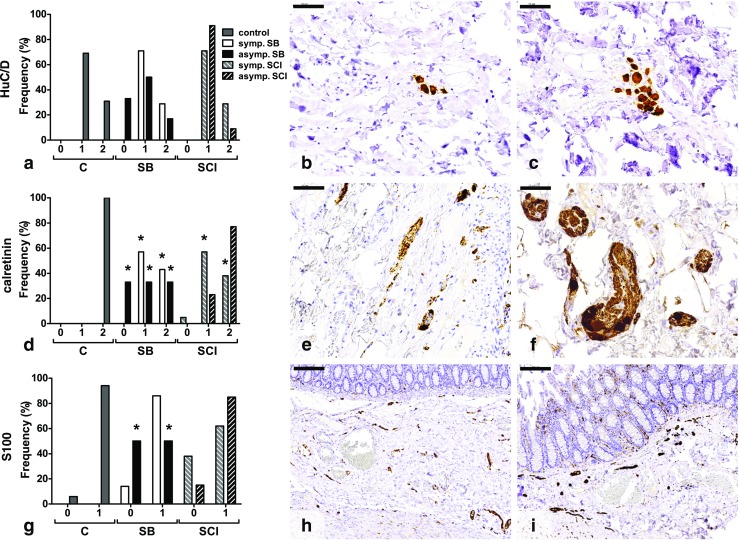
Histological findings in the submucosal plexus of colon. **a** No significant differences were found between the HuC/D-stained sections. Most tissues showed low neuronal densities (grade 1) (**b**), while in some sections, high neuronal densities (grade 2) were found (**c**). **d** Significantly less calretinin-positive neurons were found in both SB groups and the symptomatic SCI group compared with the control group. **e** Section with neurons present, but less than one neuron per neuronal structure (grade 1). **f** Minimal one neuron per neuronal structure is shown in this tissue (grade 2). **g** Low nerve fiber densities (grade 0) were most often observed in the asymptomatic SB group. **h**, **i** Illustration of low (grade 0) (**h**) and high (grade 1) (**i**) nerve fiber density. Numbers on the *x-axis* represent the semiquantitative scores. *C* control, *SB* spina bifida, *SCI* spinal cord injury. *p < 0.05 vs control. *Scale bars* 50 μm (**b**, **c**, **e**, **f**) and 200 μm (**h**, **i**)

On HuC/D staining (Fig. 1a–c), 31% of cases in the control group were scored as high neuronal density. The symptomatic and asymptomatic SB groups showed high neuronal density in 29% (*p* = 1.000) and 17% (*p* = 0.130) of patients, respectively. The symptomatic and asymptomatic SCI groups showed high neuronal density in, respectively, 29% (*p* = 1.000) and 9% (*p* = 0.350) of cases (Fig. 1a).

In the control group, at least one calretinin-positive neuron per neuronal structure was present in 100% of cases. This was significantly lower in both the symptomatic and the asymptomatic SB group (43% (*p* = 0.004) and 33% (*p* = 0.002), respectively). In SCI, only in the symptomatic subgroup was neuronal density decreased (38%, *p* < 0.001), while in the asymptomatic group, 77% of cases showed at least one neuron per neuronal structure (*p* = 0.078) (Fig. 1d–f).

The density of nerve fibers was assessed by S100 staining (Fig. 1g–i). Figure 1g demonstrates that in the control group, 94% of cases showed a high nerve fiber density. A high density was found in 86% of symptomatic SB cases (*p* = 1.000), in 50% of asymptomatic SB patients (*p* = 0.046), in 62% of symptomatic SCI cases (*p* = 0.050), and in 85% of asymptomatic SCI cases (*p* = 0.573).

#### Myenteric plexus

**Fig. 2:**
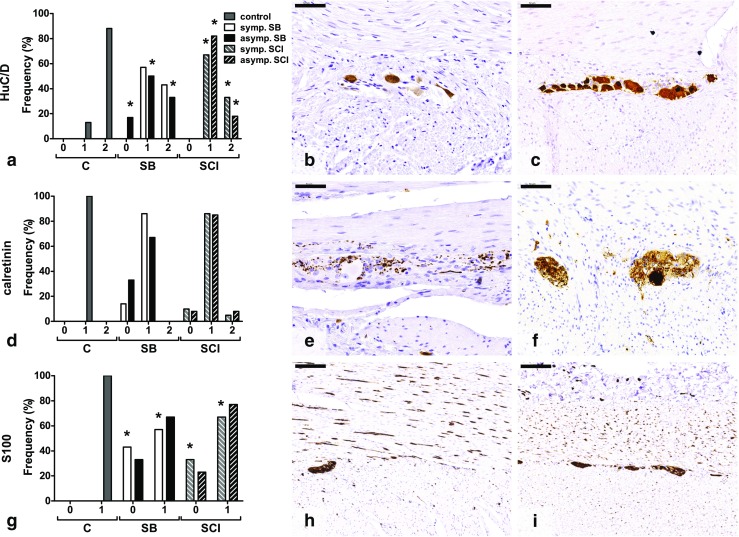
Histological findings in the myenteric plexus of colon. **a** HuC/D-positive neurons were significantly less often found in the asymptomatic SB group and both SCI groups compared with the control group. **b**, **c** Examples of low (grade 1) (**b**) and high neuronal density (grade 2) (**c**). **d** No differences in calretinin-positive neurons were found between groups. **e** Tissue with no neurons per neuronal structure (grade 0)*.* **f** Example of tissue with neurons present, but less than one neuron per neuronal structure (grade 1). **g** Lower nerve fiber densities were significantly more often found in the symptomatic SB and SCI groups compared with the control group. **h**, **i** Representation of low (grade 0) (**h**) and high (grade 1) (**i**) nerve fiber densities. Numbers on the *x-axis* represent the semiquantitative scores. *C* control, *SB* spina bifida, *SCI* spinal cord injury. *p < 0.05 vs control. *Scale bars* 50 μm (**b**, **c**, **e**, **f**) and 200 μm (**h**, **i**)

In the myenteric plexus, fewer neurons were observed in all SB and SCI groups (Fig. 2a, d).

On the HuC/D-stained sections (all neurons) (Fig. 2a–c), 88% of cases in the control group were scored as high neuronal density. In the symptomatic and asymptomatic SB groups, 43% (*p* = 0.124) and 33% (*p* = 0.025) of cases, respectively, showed a high neuronal density. The symptomatic SCI group showed only in 33% of cases a high density (*p* = 0.002), while 18% (*p* = 0.001) of the asymptomatic SCI patients showed a high neuronal density (Fig. 2a).

On calretinin staining (part of IPANs) (Fig. 2d–f), neurons were present, but less than one neuron per neuronal structure in all control cases. The symptomatic SB group showed 86% (*p* = 0.304) and the asymptomatic SB group 67% (*p* = 0.065) of cases with on average less than one neuron per neuronal structure. In the symptomatic and asymptomatic SCI groups, 86% (*p* = 0.495) and 85% (*p* = 0.192) of cases, respectively, showed presence of neurons, although less than one neuron per neuronal structure (Fig. 2d).

The S100 stain showed a lower density of nerve fibers in SB and SCI compared with the control group (Fig. 2g–i). All control cases showed a high nerve fiber density. High densities were significantly less found in the symptomatic SB group (42.9%, *p* = 0.004), while 66.7% of asymptomatic SB cases showed high densities (*p* = 0.065). In SCI, high density was significantly less often found in the symptomatic group (67%, *p* = 0.012), and 77% of asymptomatic patients showed a high density (*p* = 0.078).

**Fig. 3:**
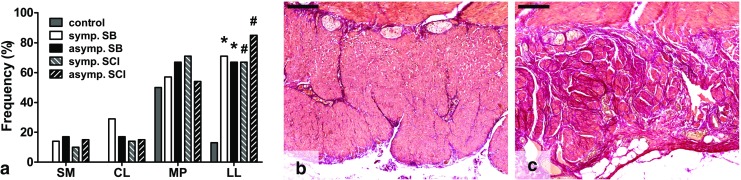
Presence of fibrosis in several layers of the bowel wall. **a** Fibrosis was significantly more often found in the longitudinal muscle layer of the SB and SCI groups. **b** Normal EVG staining pattern in the longitudinal layer of the muscularis propria (control). **c** Fibrosis in the longitudinal muscle layer, showing excessive collagen accumulation. *SB* spina bifida, *SCI* spinal cord injury, *SM* submucosa, *CL* circular layer, *MP* myenteric plexus, *LL* longitudinal layer, *EVG* elastic von Gieson. *p < 0.05 vs control, #*p* < 0.001 vs control. *Scale bars* 200 μm

The presence of fibrosis in the myenteric plexus was assessed by EVG staining. As shown in Fig. 3a, no significant differences between the control, SB, and SCI groups were found.

#### Nerve fibers within muscularis propria

In the circular muscle layer of the muscularis propria, a high nerve fiber density (S100 staining) was found in 100% of control cases. A significant decrease was seen in the symptomatic SB group (57%, *p* = 0.020), but not in the asymptomatic SB group nor in the SCI group. In the longitudinal muscle layer, no differences were observed (Suppl. Table 2).

### ICC changes

**Fig. 4:**
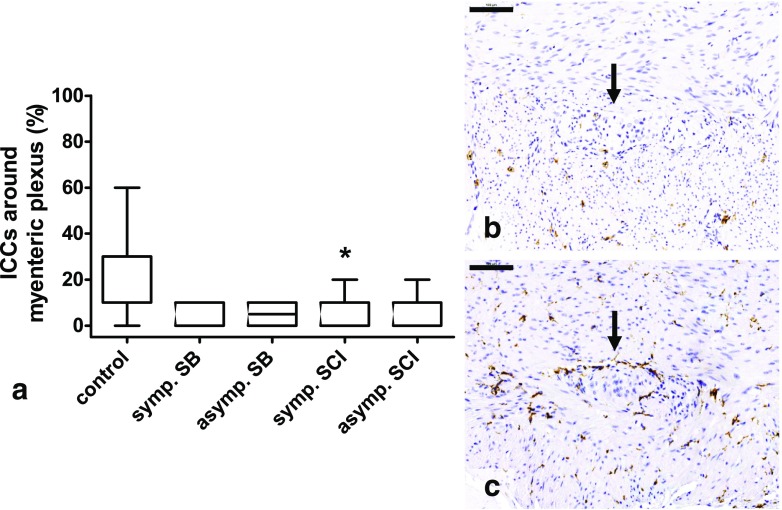
Percentage CD117-positive cells (ICCs) around the circumference of the myenteric plexus (*arrows*)*.* **a** Compared with the control group, less ICCs were found in the SB and SCI groups, significant in the symptomatic SCI group. **b** Section without ICCs around the myenteric plexus. **c** 60% of the ganglion is surrounded by ICCs. *ICC* interstitial cell of Cajal, *SB* spina bifida, *SCI* spinal cord injury. *p < 0.05 vs control. *Scale bars* 100 μm

Figure 4 shows the results of the estimation of the CD117-positive ICC network around the circumference of the myenteric plexus. Compared to the control group (mean 19.4 ± 18.1%), fewer CD117-positive cells were observed in the SB groups (symptomatic 5.7 ± 5.3%, *p* = 0.510 (*p* = 0.051 without correction for multiple testing) and asymptomatic 5.0 ± 5.5%, *p* = 0.374 (*p* = 0.037 without correction)) and SCI groups (symptomatic 5.2 ± 6.0%, *p* = 0.029 (*p* = 0.003 without correction) and asymptomatic 6.9 ± 7.5%, *p* = 0.343 (*p* = 0.034 without correction)).

### Smooth muscle changes

The smooth muscle structure of the muscularis propria was assessed using α-SMA and desmin staining. As shown in Suppl. Table 2, no significant differences were observed.

Figure 3a shows that fibrosis was not found in the circular muscle layer of the control group. Fibrosis was present in 29% (*p* = 0.083) of symptomatic and in 17% (*p* = 0.273) of asymptomatic SB patients. The symptomatic and asymptomatic SCI groups showed fibrosis in 14% (*p* = 0.243) and 15% (*p* = 0.192) of cases, respectively. The longitudinal layer showed fibrosis in 13% of control cases. Fibrosis was significantly more often found in all SB groups (symptomatic 71%, *p* = 0.011, and asymptomatic 67%, *p* = 0.025) and SCI groups (symptomatic 67%, *p* = 0.002, and asymptomatic 85%, *p* < 0.001) (Fig. 3a–c).

The presence of polyglucosan inclusion bodies in the muscularis propria was assessed by PAS staining [27, 28]. No inclusion body myopathy was found in the smooth muscle of the control, SB, and SCI groups.

## Discussion

The ENS of the colon plays an important role in the regulation of motility. Although the ENS can largely function independently, extrinsic input is needed to ensure proper bowel activity [11, 17]. The ENS is controlled by extrinsic innervation from the lower spinal cord, where parasympathetic fibers facilitate contraction of the colonic musculature and sympathetic fibers inhibit colon motility [17, 18]. Colon dysfunction following SCI can be distinguished into two main types, dependent on the level of the lesion: an upper motor neuron syndrome and a lower motor neuron syndrome [4, 29]. The upper motor neuron bowel is associated with spastic segmental contractions of the colon and decreased propulsive peristalsis, while the lower motor neuron bowel is associated with areflexia and reduced colon motility. Both syndromes result in constipation [29].

Disruption of the extrinsic nerve fibers as in patients with SB and SCI results in changed ENS activity and subsequently in impaired motor function of the bowel [1, 8, 20, 30]. We expected histological changes in the intrinsic neuromuscular apparatus of the colon, due to impaired extrinsic control. Main alterations were expected in the myenteric plexus, which controls motility [10, 11]. Histological evaluation of the neuromuscular structures (ENS, ICCs, smooth muscle) may provide insight in the pathophysiological mechanisms of neurogenic bowel in SB and SCI.

In the current study, we observed four changes in the neuromuscular apparatus. First, in analogy with two studies that investigated the effect of spinal cord and (parasympathetic) peripheral nerve lesions on the ENS, we did find loss of ganglion cells in the myenteric plexus of the colon. However, the submucosal plexus was considered to be normal [21, 31], which we confirmed in our study. Upon analysis of subsets of neurons, we observed loss of calretinin-positive neurons in the patient groups. Calretinin is a biomarker for a subpopulation of neurons including IPANs [32]. IPANs are sensory neurons which play a role in the peristaltic reflex [33]. In contrast, in the myenteric plexus, a reduction of the total neuron population was observed, both in the symptomatic and asymptomatic SB and SCI groups, which suggested that neuron loss could thus not solely be responsible for severe motility complaints.

Second, we found lower fiber density in the symptomatic but not in the asymptomatic groups. Since enteric glial cell nuclei are more prominent than nerve fibers in the healthy myenteric plexus, we believe that glial cells are lost in the symptomatic groups. Although the exact function of glial cells in intestinal motility is still unclear, they may have an important role in the physiological control of motility and in enteric neurotransmission [13, 34]. Loss of enteric glial cells might impair bowel motility, as has been shown both in humans [35] and mice [36]. Our findings provide supportive evidence for a significant role of glial cells in the regulation of motility. Whether reduction of nerve fibers initiates neuronal loss or is a response to neuronal loss remains still unclear; further research on this mechanism is needed.

Third, we found loss of the ICC network around the circumference of the myenteric plexus in almost all patients, although after performing a strict statistic test (Kruskal-Wallis) with correction for multiple testing, this was only significant in the symptomatic SCI group. The small subgroups resulted in low statistical power and lack of significance. Scoring of the density of the ICC network has shown good inter- and intraobserver agreement and reliability [26], but the differences between our groups might be a result of rater variability. A previous study indicated that only tissues with completely absent ICCs may be diagnosed as abnormal in the colon [26]. Several studies have shown that loss of ICCs in patients with motility problems is highly associated with changes of enteric nerves [37]. Consequently, in most motility disorders, it is unclear whether ICC network disruption is primary or secondary to neuronal changes [38]. We hypothesize that in our patients, ICC loss is indeed secondary to disruption of the extrinsic innervation in SB and SCI. Loss of the myenteric ICC network was shown in most patients, while approximately two thirds of this group revealed neuronal loss. Therefore, disruption of the ICC network may precede loss of enteric neurons.

Fourth, significantly more fibrosis was found in the longitudinal muscle layer in almost all patients. Although fibrosis in the longitudinal layer is frequently found in patients with visceral myopathy [23, 39, 40], other muscle changes were not observed in our patients. Nevertheless, the presence of fibrosis might contribute to motility problems.

Despite SB being a congenital and SCI an acquired disease, no clear differences between both conditions were found. All observed alterations in the neuromuscular apparatus may be explained as reactive to disrupted extrinsic innervation of the colon. This is supported by a study in rats with myelomeningocele, suggesting that the anorectal unit is normally developed [41]. This has led to the hypothesis that loss of extrinsic innervation may result in trans-neuronal degeneration, although the exact mechanism in the bowel is currently unknown [21, 31].

Our study has some limitations. First, we aimed to study two large groups of patients to perform reliable comparisons which required collection of tissue blocks from SB and SCI patients on a national scale. We used proper cross sections of the entire colonic wall, from mucosa to serosa, and included cross sections in different directions to make the groups as large as possible. Nevertheless, the groups, notably the SB groups, were relatively small which resulted in low statistical power. Second, the anonymous character of the study resulted in lack of data on the severity of motility problems, the level of the lesion, the duration of the SCI, mobility of patients (bedridden, use of wheelchair), and medication. Patients in the symptomatic SB and SCI groups had severe motility problems, which was the indication for surgery. In contrast, of patients in the asymptomatic SB and SCI groups, it remains unknown whether or not motility was normal. These patients might have suffered from motility problems in less severe form, which would be supported by the histological changes in these groups. Third, the mean age in the SB groups was much lower than that in the control group. Therefore, differences in enteric neuronal numbers between controls and SB patients might be underestimated as patients in the control group might have lost neurons due to aging [42].

In conclusion, we have shown that impaired extrinsic innervation affects neuromuscular structures which may contribute to decreased bowel motility. Major neuromuscular alterations in the colon of SB and SCI patients are loss of myenteric neurons and disruption of the ICC network around the myenteric plexus, accompanied by decreased nerve fiber density in the myenteric plexus. These changes might affect intestinal motility, already symptomatic in some patients and potentially symptomatic in others. In addition, nerve fiber density (glial cells) in the myenteric plexus was significantly decreased in the symptomatic SB and SCI groups, which may be the reason for the major motility problems found in these patients.

## Electronic supplementary material

ESM 1 (PDF 311 kb)

ESM 2 (PDF 7235 kb)

ESM 3 (PDF 2526 kb)

ESM 4 (PDF 975 kb)

ESM 5 (PDF 805 kb)

## Abbreviations

CNS: Central nervous system
ENS: Enteric nervous system
EVG: Elastic von Gieson
GINMD: Gastrointestinal neuromuscular disease
H&E: Hematoxylin and eosin
ICC: Interstitial cell of Cajal
IPAN: Intrinsic primary afferent neuron
PAS: Periodic acid Schiff
SB: Spina bifida
SCI: Spinal cord injury
SMA: Smooth muscle actin
