# Supplementary material for: The enteric nervous system and the musculature of the colon are altered in patients with spina bifida and spinal cord injury
Source: Virchows Arch. 2017 Jan 6;470(2):175–84. doi: 10.1007/s00428-016-2060-4 (PMC5306076; doi:10.1007/s00428-016-2060-4)
Supplement: Supplementary file 1 — (PDF 311 kb) [file 428_2016_2060_MOESM1_ESM.pdf]

Neuromuscular changes in the colon in spina bifida and spinal cord injury: a nationwide histology study

Corresponding author: Marjanne.denBraber-Ymker@radboudumc.nl
Virchows Archiv

Suppl. Table 1 Overview of the main conclusions. Differences compared with the control group are represented as no difference (=), decrease/increase (↓/↑), or significant decrease/increase (↓↓/↑↑)

	SB		SCI	
	symptomatic	asymptomatic	symptomatic	asymptomatic
**Submucosal plexus**				
Total neurons (HuC/D)	=	=	=	↓
Calretinin positive neurons	↓↓	↓↓	↓↓	↓
Nerve fibres	=	↓	↓	=
**Myenteric plexus**				
Total neurons (HuC/D)	↓	↓	↓↓	↓↓
Calretinin positive neurons	=	↓	=	=
Nerve fibres	↓↓	↓	↓↓	↓
ICC network	↓	↓	↓↓	↓
**Circular muscle**				
α-SMA	↓	=	=	=
Desmin	=	=	=	=
Fibrosis	=	=	=	=
Nerve fibres	↓↓	=	=	=
**Longitudinal muscle**				
α-SMA	↓	=	=	=
Desmin	=	=	=	=
Fibrosis	↑↑	↑↑	↑↑	↑↑
Nerve fibres	=	=	=	=

α-SMA, α-smooth muscle actin; ICC, interstitial cell of Cajal; SB, spina bifida; SCI, spinal cord injury

Neuromuscular changes in the colon in spina bifida and spinal cord injury: a nationwide histology study

Corresponding author: Marjanne.denBraber-Ymker@radboudumc.nl

Virchows Archiv

Suppl. Table 2 Semiquantitative evaluation of immunohistochemical markers in the different layers of colonic muscularis propria in controls and in patients with spina bifida (SB) or spinal cord injury (SCI). Expression of α-SMA is more often lower in the circular and longitudinal muscle layer of the symptomatic SB group (not significant). In the circular layer, lower nerve fibre densities (S100) are most frequently found in the symptomatic SB group

		Control	SB				SCI			
			symptomatic		asymptomatic		symptomatic		asymptomatic	
**Antibody**	**Score**	n (%)	n (%)	p-value	n (%)	p-value	n (%)	p-value	n (%)	p-value
α-SMA CL	0	1 (6.2%)	3 (42.9%)	.067	0 (0%)	1.000		.432		1.000
	1	15 (93.8%)	4 (57.1%)		6 (100%)		21 (100%)		12 (100%)	
α-SMA LL	0		2 (28.6%)	.083	0 (0%)					
	1	16 (100%)	5 (71.4%)		6 (100%)		21 (100%)		123 (100%)	
desmin CL	0									
	1	16 (100%)	7 (100%)		5 (100%)		17 (100%)		13 (100%)	
desmin LL	0		0 (0%)		1 (20.0%)	.238	0 (0%)		1 (7.7%)	.448
	1	16 (100%)	7 (100%)		4 (80.0%)		17 (100%)		12 (92.3%)	
S100 CL	0		3 (42.9%)	.020*	0 (0%)		2 (9.5%)	.495	1 (7.7%)	.448
	1	16 (100%)	4 (57.1%)		6 (100%)		19 (90.5%)		12 (92.3%)	
S100 LL	0	15 (93.8%)	5 (71.4%)	.526	6 (100%)	1.000	17 (81.0%)	.364	11 (84.6%)	.573
	1	1 (6.2%)	2 (28.6%)		0 (0%)		4 (19.0%)		2 (15.4%)	

α-SMA, α-smooth muscle actin; CL, circular layer; LL, longitudinal layer; SB, spina bifida; SCI, spinal cord injury. *$p<0.05$ vs control
