# Supplementary material for: The enteric nervous system and the musculature of the colon are altered in patients with spina bifida and spinal cord injury
Source: Virchows Arch. 2017 Jan 6;470(2):175–84. doi: 10.1007/s00428-016-2060-4 (PMC5306076; doi:10.1007/s00428-016-2060-4)
Supplement: Supplementary file 2 — (PDF 7235 kb) [file 428_2016_2060_MOESM2_ESM.pdf]

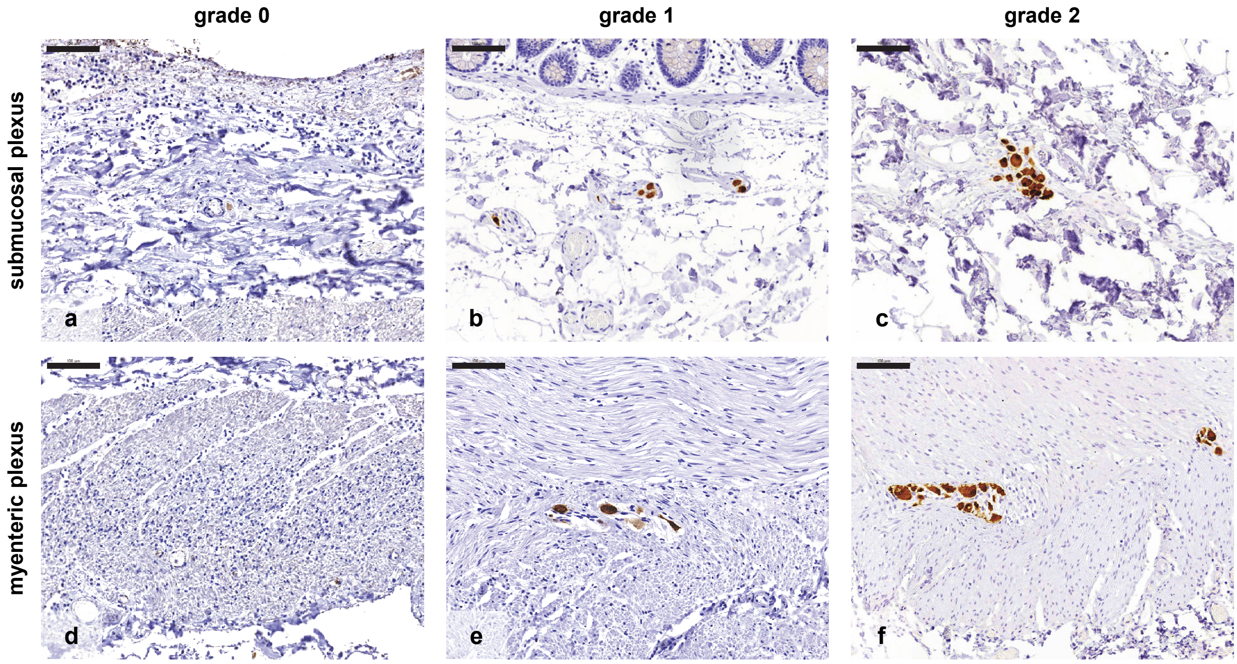

Suppl. Fig. 1
Semiquantitative scoring of HuC/D stained sections. The number of neurons in relation to the present plexus was estimated in HuC/D sections as follows: Firstly, the distribution of the neuronal network was evaluated on S100 stained sections. Subsequently, the number of neurons per ganglion was estimated in the HuC/D staining in relation to this neuronal network and scored as no neurons (0), low neuronal density (1), and high neuronal density (2). *Scalebars* 100 µm

Neuromuscular changes in the colon in spina bifida and spinal cord injury: a nationwide histology study

Corresponding author: Marjanne.denBraber-Ymker@radboudumc.nl
Virchows Archiv
