# Supplementary material for: The enteric nervous system and the musculature of the colon are altered in patients with spina bifida and spinal cord injury
Source: Virchows Arch. 2017 Jan 6;470(2):175–84. doi: 10.1007/s00428-016-2060-4 (PMC5306076; doi:10.1007/s00428-016-2060-4)
Supplement: Supplementary file 3 — (PDF 2526 kb) [file 428_2016_2060_MOESM3_ESM.pdf]

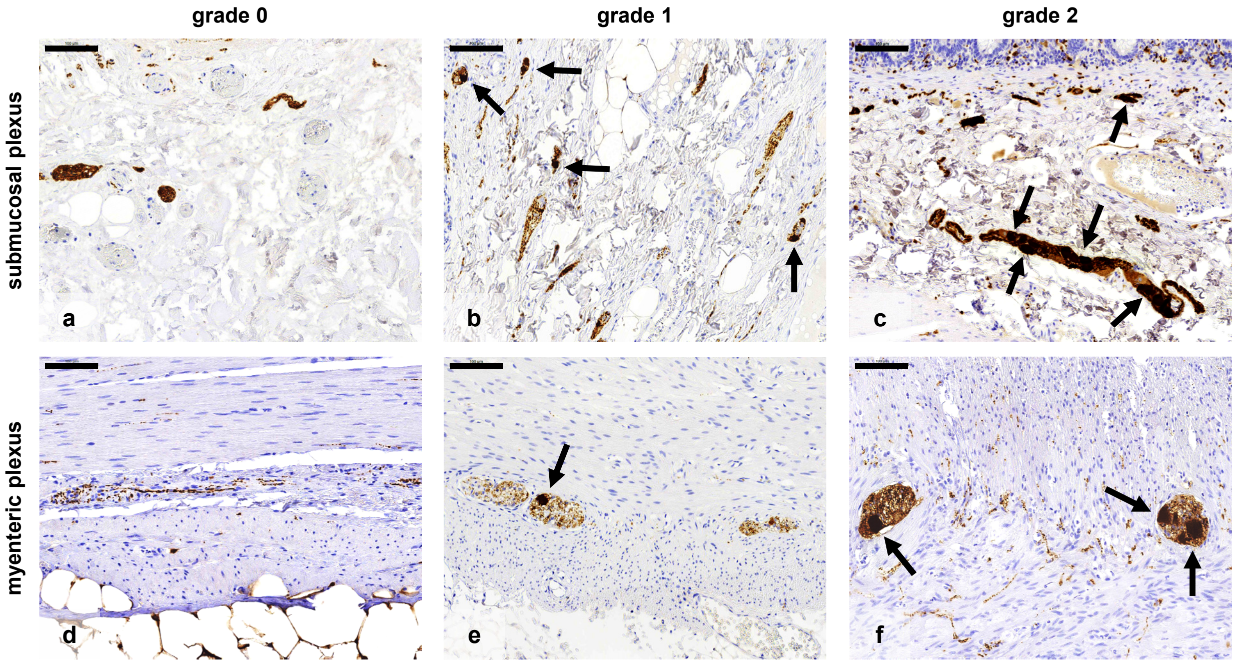

Suppl. Fig. 2
Semiquantitative scoring of calretinin stained sections. The number of neurons was estimated in relation to the present plexus: no neurons (0), on average less than one neuron per neuronal structure (1), minimal one neuron per neuronal structure (2). Neurons are indicated with *arrows*. *Scalebars* 100 µm

Neuromuscular changes in the colon in spina bifida and spinal cord injury: a nationwide histology study

Corresponding author: Marjanne.denBraber-Ymker@radboudumc.nl
Virchows Archiv
