# Supplementary material for: The enteric nervous system and the musculature of the colon are altered in patients with spina bifida and spinal cord injury
Source: Virchows Arch. 2017 Jan 6;470(2):175–84. doi: 10.1007/s00428-016-2060-4 (PMC5306076; doi:10.1007/s00428-016-2060-4)
Supplement: Supplementary file 4 — (PDF 975 kb) [file 428_2016_2060_MOESM4_ESM.pdf]

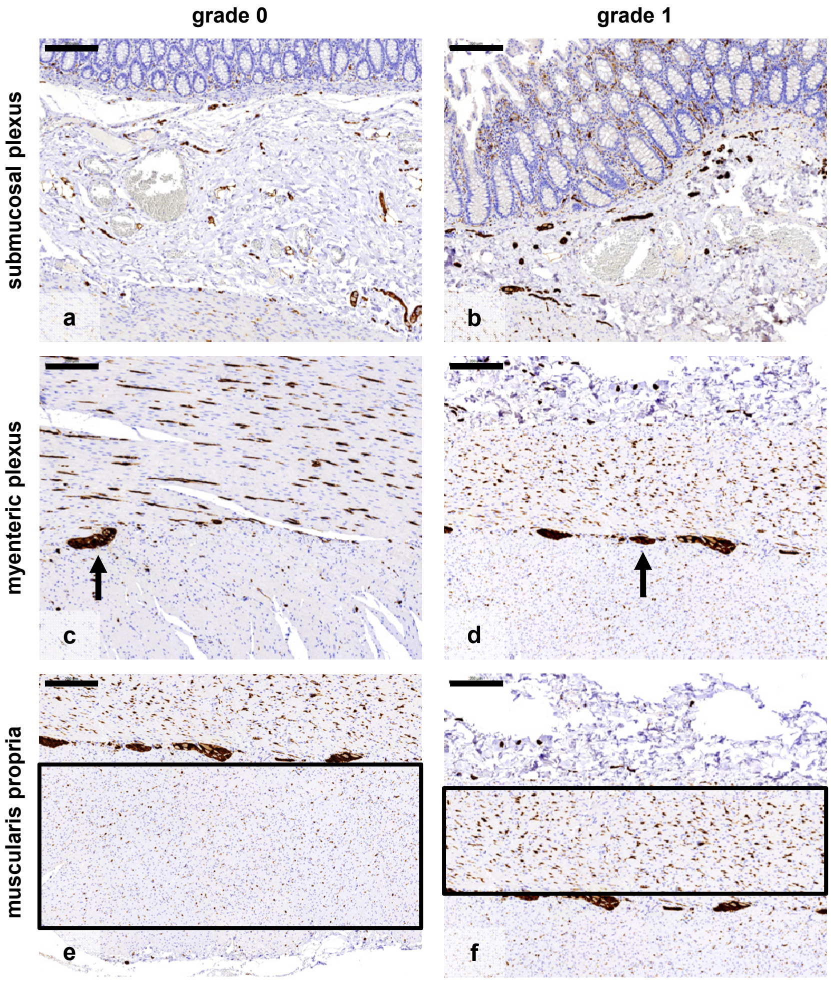

Suppl. Fig. 3
Semiquantitative scoring of S100 stained sections. The density of nerve fibres was assessed in the submucosal plexus (**a, b**), the myenteric plexus *(arrows)* (**c, d**) and the muscularis propria (area within *rectangle*) (**e, f**) as follows: no or low density (0) and high density of S100 positive fibres (1). *Scalebars* 200 µm

Neuromuscular changes in the colon in spina bifida and spinal cord injury: a nationwide histology study

Corresponding author: Marjanne.denBraber-Ymker@radboudumc.nl
Virchows Archiv
