# Supplementary material for: The enteric nervous system and the musculature of the colon are altered in patients with spina bifida and spinal cord injury
Source: Virchows Arch. 2017 Jan 6;470(2):175–84. doi: 10.1007/s00428-016-2060-4 (PMC5306076; doi:10.1007/s00428-016-2060-4)
Supplement: Supplementary file 5 — (PDF 805 kb) [file 428_2016_2060_MOESM5_ESM.pdf]

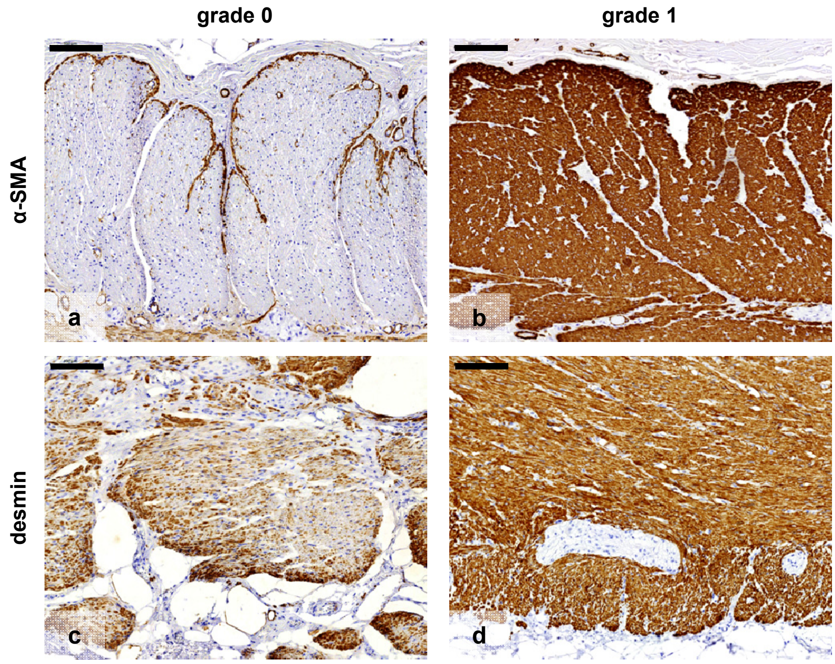

Suppl. Fig. 4
Semiquantitative scoring of α-Smooth muscle actin (SMA) (**a**, **b**) and desmin (**c**, **d**) stained sections. Staining intensities were scored as follows: no or weak staining (0) and strong staining intensity (1). Internal references for α-SMA and desmin were respectively immunoreactivity within the blood vessel wall and muscularis mucosae (1). *Scalebars* 100 µm

Neuromuscular changes in the colon in spina bifida and spinal cord injury: a nationwide histology study

Corresponding author: Marjanne.denBraber-Ymker@radboudumc.nl
Virchows Archiv
